# Therapeutic Sociality: Empathy and care amongst distressed Ethiopian domestic workers

**DOI:** 10.1111/etho.70041

**Published:** 2026-08-31

**Authors:** Eva Melstrom

**Affiliations:** Department of Comparative Human Development, The University of Chicago, Chicago, Illinois, USA

## Abstract

Ethiopian migrant domestic workers frequently experience violence and abuse during employment, which can lead to serious emotional and mental distress. After returning to Ethiopia, some distressed women receive care at residential facilities specifically dedicated to rehabilitating returned domestic workers. In this paper, I examine the narratives from previously distressed domestic workers living in one such facility to understand how they view and respond to the distress of newly arrived returnees. I suggest that through embodied knowing they position the returnees’ distress as familiar, and, importantly, as ambiguous and temporary. It is at this intersection of ambiguous-familiarity where I situate an ethics of care marked by an empathic understanding of having-been-there and what-will-be, a past and future oriented knowledge that precipitates caring responses marked by calm, gentle, and ordinary engagements—a therapeutic sociality. I demonstrate that while empathy may be central to other therapeutic communities across various histories and geographies, it is a foregrounding of embodied knowledge and the imaginative projection of what-will-be amongst previously distressed returned Ethiopian migrant domestic workers that is distinctive to their ethics of care, and may offer both a practical and ethical resource for rehabilitating returned domestic workers.

Zeynab worked in the United Arab Emirates for four months before her “sickness” appeared. The exact details of when it started are vague, but she does remember her employer bringing her back to the *delala*^1^ — the person who oversaw her transnational migration and employment. She likewise cannot recall her return flight to Ethiopia nor her arrival to Tesfa^2^, a residential home in Addis Ababa that provides care for returned domestic workers. Yet, she clarifies, she does have some recollection of “running around” and “hearing some kind of voices.” She felt as if everyone was talking about her.

“I was scared. I wanted to kill myself,” she explains.

“I used to be like Hannah, but worse. I imagined other people. I ate dirt.”

I look over at Hannah. She is currently sauntering around the compound. A few weeks prior, Hannah approached me to compare our features. Using the English, rather than the Amharic name, she tells me that hers are similar to “Lucy’s,” the 3.2-million-year-old *Australopithecus afarensis* discovered by paleoanthropologist Maurice Taieb during a 1974 field expedition in the Afar region of Ethiopia.

“I am the Ethiopian Lucy. I am beautiful, but people don’t give me respect.” Her eyes appeared teary. My research assistant, Abinet, and I confirm our respect for her and agree with her aesthetical self-assessment. She seems satisfied and moves on.

This was not the first time that Hannah offered us information about herself that was ostensibly untrue—that is, she was not *the* Lucy—yet, like her fellow residents, Abinet and I made no objections. Determining the truthfulness of her words did not matter to us, nor, in my observations, to her cohabitants. Whether it was her claims of multiple homeownerships or the impending return of her American boyfriend, I never witnessed her fellow residents rebuff or reject her stories. Instead, they made conversation with her in a manner of curious or playful regard. She was socially engaged with gentle care.

The guard turns his attention to us and interjects, speaking to Zeynab with a smile, “Do you remember rolling around on the ground, in front of the gate?”

Zeynab seems a bit embarrassed, and nods in agreement, “I wanted out.”

She explains that during that time she also removed her clothes and took everyone’s food.

“That is why she is fat!” one woman jests.

In the midst of our conversation, a new returnee approaches. She has been at Tesfa for a day and has yet to speak. Pointing to the slashes through her eyebrows, one of the women remarks that she thinks this newcomer is from the Tigray region. The newcomer remains quiet.

Another woman tries to speak with her, “Endemin-nesh?” (How are you?)

No response. Someone else offers her a chair. She silently accepts and sits down. Then, Zeynab makes an interesting remark. She tells me that while many women who come through Tesfa are sick, “there are no people that are as sick as me.”

She continues, “And now that I am better, I watch the sick ones. I observe them. I recognize what they are doing. I know their feeling.”

She pauses. “But soon, they will calm down.”

“How do you know this?” I ask.

Abinet translates my question and Zeynab’s response, “She says she knows this because she did.”

## INTRODUCTION

In the presence of Hannah and her probable fictitious claims, and the newcomer’s muted approach, how might we understand Zeynab and her cohabitant’s remarks and comportments? Are Zeynab’s partial recollections about her own sickness and declared knowledge about other returnees’ sicknesses—varied assemblages of symptoms and behaviors—advantageous or trivial? Do residents of Tesfa care about the wellbeing of distressed newcomers, or are they indifferent, unmoved by what they see as renderings of their own former sickness? Might a simple greeting or an offer of a place to sit be understood as more than mere pleasantries, and instead, as indicative of an empathically grounded ethics of care that is efficaciously attentive to the needs of another?

In this article, I take up these questions and offer insights garnered from individuals who endured emotional and mental distress, what they call “sickness,” in relation to their experiences as migrant domestic workers to demonstrate how their recognitions of self-sameness in distressed new returnees may be thought of as caring, and revealing of a locally situated form of “marked empathy” (Hollan & Throop 2008). That is, the embodied and articulated knowing of another’s condition and the corresponding intersubjective dynamics within Tesfa demonstrate a specific form of empathic care. In taking this stance, I situate empathetic responses within Tesfa as embedded within a distinct social and interpersonal context involving a form of discernment that is constituted by a having-been-there and a future-oriented what-will-be unique to returned Ethiopian migrant domestic workers. Below I illustrate how this experiential and embodied way of understanding mental and emotional distress is based in empathy and can precipitate an efficacious form of care within Tesfa, what I call therapeutic sociality—a way of being with one another that is marked by calm, gentle, and commonplace engagements.

In examining situational, and conceivably, cultural processes of empathy and care within Tesfa, through a feminist phenomenological logic, I offer a viewpoint distinct from the biomedical psychiatric discourses and practices frequently imposed upon Ethiopian domestic workers in their home and host countries, which tend to stipulate that they suffer from high rates of discernable psychiatric diseases and need pharmaceutical intervention (Aziz et al. 2023; Zeleke et al. 2015). A feminist phenomenological perspective gives pause to this claim by first turning to the experiences and narratives of my interlocutors to revise and reorient our representations of and responses to decrees of otherness (i.e., abnormality and deviance). It places the onus of knowledge on returned domestic workers’ renditions of what causes, constitutes, and heals their own and their compatriots’ distress, and upholds ambiguity as a way to unsettle claims of certitude (Beauvoir 2018; 2011; Melstrom 2026). It allows for an examination into distress, as experienced by returned Ethiopian domestic workers, less as a distinct brain disease and more as a protean byproduct of multiple and interacting situations, bringing into relief how women in Tesfa are uniquely situated and informed beings who understand what constitutes restorative, ethical, and good care. In other words, this practice of thinking and analyzing reframes the body as a temporal situation that is shaped in relation to its social world and consequently comes to embody a dynamic experiential knowledge.

This theoretical and methodological stance gives weight to how women of Tesfa situate their own and their compatriot’s distress as “sickness”—an ambiguous and temporary state. It is my interlocutor’s use of the words “*beshta*” (disease/sickness) “*tamime*” (while I was sick), and “*amemegn*” (I am sick), in reference to their own and other’s distress, which work as nebulous catch-alls for a range of behaviors, from incoherent speech to memory loss, delusions to catatonia, as well as physical ailments such as headaches and fatigue. This ambiguous construal is a practice that allows healed women to imaginatively project a meaningful knowledge about “sickness.” It is an imprecise way of conceptualizing and containing conditions that avoids more stigmatized/stigmatizing mental illness classifications such as *“ye aemro beshitegna/ hmemtegna”* (mental patient), “*edbe*” (mad or crazy), or *“gemash Amanuel”—*a term that incorporates the well-known psychiatric hospital in Addis Ababa to signify that one is “crazy” (Girma et al. 2024)—while also allowing them to recognize elements of their own experience in the other.

Furthermore, their use of the term “sickness” is, I suggest, indicative of the ordinary positioning of distress within the context of returned domestic workers (Kaiser et al. 2015; Kaiser & Weaver 2019; Nichter 1981; Snodgrass et al. 2023). By situating expressions of distress as sickness, my interlocutors are implying that such expressions among returnees are unexceptional—that is, they are recognized as personally familiar and experientially and emotionally accessible; and accordingly, understood as normal and ordinary reactions to situations of domestic work abroad. A sentiment underscored by Zeynab, and others discussed below.

By positioning sickness as an unexceptional and ordinary condition, it may also be thought of as a complementing counterpoint to extraordinary conditions (Jenkins 2015), in that the conditions my interlocutors experience and remark upon encapsulates mental and emotional states that are often defined by psychiatrists within Ethiopia as mental illnesses resulting from their experiences as domestic workers (Desie et al., 2024; Getnet et al. 2016; Tilahun et al. 2020), and yet, as Zeynab’s statement indicates, the “nonordinary and spectacular qualities of mental illness in experience and representation” (Jenkins 2015, 1)—hearing voice, delusions of reincarnate hominis, pica—are in fact ordinary and nonstigmatized given their prevalence and personal familiarity within Tesfa.

In exploring how women in Tesfa embody, speak about, and respond to distress this article, then, has three objectives: Primarily, this work seeks to contribute to conversations on the benefits of therapeutic communities (TC) and the relational process of care by focusing on a women’s residential home in Ethiopia—a social, cultural, and experiential context absent from current discussions. To achieve this end, I juxtapose Tesfa to two psychiatric communities in distinct geographical and historical spaces to, first, consider how empathy has been fundamental to the ethics of care within specific TC and, second, to illustrate how it is the foregrounding of an embodied and temporally-oriented knowing—a feeling of having been-there and imagined what-will-be—that is unique to Tesfa residents and their local variety of empathic care.

The second aim of this paper is more theoretical. In using a feminist phenomenological methodology to establish how residents of Tesfa express a knowledge of how best to encounter another’s distress, this theoretical and ethnographic mediation is a means toward expanding our thinking about the relationship amongst sociality, empathy, and care by disrupting “sedimented” conversations about feelings, practices, and experiences associated with the relational production of empathy and ethical care (Pedwell 2016; Fielding 2017; Mattingly 2014; Melstrom 2026). This feminist stance underpins my claim that the encounters within Tesfa, which are seemingly marked by alterities that might be regarded as impenetrable to “direct” perception and therefore not conducive to empathic understanding [i.e., the expressed distress is so bizarre an onlooker could not possibly know what the other is experiencing and thus could not be “experientially acquainted” (Throop & Zahavi 2020, 289)], *are* in fact open to empathic understanding precisely *because* the distress is regarded as ambiguous-yet-familiar via an imagined shared experience of sickness in the context of domestic work.

For instance, women in Tesfa did not need to be struck by the same employer, in the same way, to understand each other’s fear, pain, or disillusionment; nor did they need to experience the same delusions or rage to understand each other’s sickness; and, importantly, empathic understanding was not necessarily facilitated by the narrative release or the verbal exchange of analogous experiences, techniques familiar to other modes of healing in relation to trauma (Young 1997; McKinney 2008). On the contrary, I was told by several interlocutors that they did not want to think or dwell upon what happened to them; they did not want to give space to those thoughts. “Too much thinking” or “deep thinking” as Taytu, whom I discuss below, said, could cause “*chiniket*” (stress)—one of the triggers of sickness. Therefore, the exactitudes of one’s experience did not appear paramount for empathic understanding; rather, it was the “felt sense” (Gendlin 1964) of having been there; a recognition of a shared lived experience shaped by specific forces of the exterior world (Fielding 2017).

This interpersonal understanding relates to a third aim, or perhaps part “b” of my second aim. In offering a space to think about how empathy emerges in Tesfa and serves as an ethics of care for residents, this work contributes to discussions about the muddied “appellations of care” and the interactional contingencies of caring relationships (McKearney 2020). Specifically, I situate therapeutic sociality as a practice of best “good care” as it considers the enmeshment of one’s social environment, individual experiences, and ethical ways of caring-for (Mattingly 2014). This paper, then, offers both practical and theoretical extensions concerning what ethical and good care looks like in murkier contexts.

## SETTING AND CIRCUMSTANCES

Tesfa is an Ethiopian non-governmental organization (NGO) whose stated objective is to rehabilitate and reintegrate returned domestic workers, tens of thousands of whom return each year (Adugna 2022). The women who reside at Tesfa are part of a significant—though relatively recent—cultural practice of journeying to the Arabian Peninsula and Levant countries for domestic work, which is characterized by experiences of both subjugation and success (Anbesse et al., 2009; Fernandez 2020; Fernandez & de Regt 2014; de Regt & Tafesse 2016; Shewamene et al. 2022). The experiences of the women with whom I spoke align with the former.

The majority of women brought to Tesfa are done so following their arrival to Bole International Airport in Addis Ababa, Ethiopia’s capital. They are most often identified by an airport-employed nurse—regularly stationed near baggage claim or an arrival gate—who determines that a woman is “lost,” a label bestowed upon someone if they cannot identify the location of their home village or know how to contact their family. The airport nurses may also identify someone as “mentally ill,” which is determined through such symptoms as shouting, delusions, muteness, or assaults against those nearby.

Less frequently, a woman may be identified by a staff member from the International Organization for Migration (IOM) working inside or outside the airport. There was one dedicated employee who positioned himself around the airport parking lot to identify returned domestic workers who were unsure of their surroundings. He would approach them and offer assistance. Sometimes it was welcomed and other times not. Afterward, whether it be an airport nurse or an IOM officer, a request may be made to the Ethiopian Red Cross Society for transportation to Tesfa, or less frequently, a phone call to the psychiatric nurse for a pickup. If a new returnee arrived on a weekday morning, there was a good chance she would encounter the psychiatric nurse. Otherwise, she would be dropped at the front gate, allowed entrance by the guard, and greeted only by other returnees.

As both a short- and long-term residential facility, Tesfa can at times feel transitory. Over the course of my ten months of fieldwork, the number of residents fluctuated between three to as many as ten, though ten would have been uncommon. On average, one or two new returnees arrived each week, often under duress and in distress, while a similar number of women departed for home. There was not a standard amount of time women resided at Tesfa. However, the stated goal was to rehabilitate women (who had been diagnosed by the nurse with a psychiatric condition such as depression, anxiety, or posttraumatic stress disorder) over the duration of three months with prescribed pharmaceuticals. This was rare. It was more common for an individual to make contact with a family member and subsequently depart after a few weeks. Many departed after only six or seven days. Some with a three-month supply of risperidone, most with nothing.

During their time at Tesfa, residents mainly spent their days talking, sleeping, cooking, playing cards, making coffee, washing laundry, and watching television. For those who remained longer than a week, routines inevitably included the arrival of a returnee exhibiting distress. Such arrivals, as this article discusses, generally elicited little disruption to rhythms of daily life (though not exclusively).

Upon entering Tesfa, through a locked, metal gate, one is welcomed into a spacious courtyard. To the left stands a large, white tent embossed with the emblem of a recognizable global humanitarian aid organization. A rectangular table and a handful of scattered plastic chairs rest underneath. To the right hangs an old and fragmented basketball hoop. At the back of the courtyard stands the two-story residence. Fashioned from concrete, steel, and glass, it is a relatively modern facility by both local and international standards. The main floor consists of a large room comprised of six sets of bunk beds and a small television suspended in the corner; a toilet room is just thru the corridor. Toward the rear of the house stands a fully equipped kitchen.

On the second floor there is an administrative office adorned with hundreds of photos of former residents reuniting with family members. Across the hallway is the nurse’s examination room, which holds a small cot, a table, and two chairs. This space also includes a built-in counter that incorporates lockable drawers. One secured drawer contains small vials of haloperidol and risperidone. At the end of the hallway there is a small room containing a solitary bed. I was told that this space was reserved for special circumstances. I saw it occupied only once by a woman and her infant daughter, both of whom showed signs of severe malnourishment.

The bodily inscriptions of abuse amongst residents extend beyond that of starvation and traumatic childbearing incidents. My interlocutor’s experiences of violence (including verbal, physical, and sexual assault, isolation and confinement, overwork, underpayment and unpayment, and inadequate nutrition and hydration) match those well-documented by both human rights groups and scholars examining Ethiopian migrant domestic workers in the Gulf States, Israel, and Lebanon (Amnesty International 2019; de Regt & Tafesse 2016; Demissie 2018; Fernandez 2020). Despite the infamy and ubiquity of violence amongst Ethiopian migrant domestic workers, I hesitate to give vivid accounts. I continue to grapple with the ethics of this type of anthropological project, and I feel uneasy about sharing another’s story of abuse for academic rigor or gains. I question the value I add with explicit details of bodily and psychic harm: How much detail is enough to validate my claims and do justice to those who shared their stories? Is it not noteworthy that many interlocutors focused on the immorality and cruel nature of their employers, rather than the abuses that were done to them? I heard more than once that it was their beauty, as Ethiopian women, that triggered the violent jealousy in their employer.

Additionally, while the majority of interlocutors did not refuse to share such stories with Abinet and me, some did refuse to speak to another researcher and an international journalist. I interpret this decision in a couple of ways. One explanation recognizes this refusal as a politics of partiality; it mattered who could hear their stories (Simpson 2007). Though I can only speculate how such mattering was determined, it likely had something to do with the consistency of my presence—the aforementioned others dropped in and out. Many women observed me, as I observed them; some inquired about my life, asking about my nationality, religion, and whether I had children. They had access to me in a way that they did not have access to other outsiders. Yet, there was one personal characteristic that I credit with granting me the ability to form connections, trusting relations, and a relatability that ostensibly eclipsed our many differences: I was deeply shy.

I never imagined my research would take me 7000 miles from my home. I grew up in a small, rural town in the midwestern United States. My interest in mental health began there, as a 14-year-old, after one of my dearest friends died by suicide. I did not want his death to be in vain; I promised myself I would do good in his memory. From undergraduate to graduate research, I maintained my focus on the subject of suicide, a topic that eventually brought me into contact with members of the Ethiopian diaspora in Boston—they had experienced their own high rates and wanted to understand why (Melstrom 2014). Throughout the next four years of fieldwork, I listened to members of the community and took seriously their advice that I *needed* to go to Ethiopia to “really understand” their explanations and insights. I obliged. I followed the object in order to trace the cultural formation of suicide and depression in the context of Ethiopia and movement abroad (Marcus 1995, 96). So, here I was, after another series of unexpected trajectories, sitting at Tesfa, trying to be as inconspicuous as possible.

While not necessarily valuable to all of my fieldwork activities, within the environment of Tesfa, being reserved worked in my favor. Abinet explained to me that my shyness was a sign of respect; it was a cherished Ethiopian trait that was part of the “tradition.” Furthermore, my shyness was often accompanied by obvious blushing, which became a source of entertainment.

“Eevayee!” (“my dear Eva”), the nurse would declare, as she boisterously laughed alongside the other residents at my poor attempts to converse in Amharic or consume an improperly folded morsel of *injera* and meat. I was embarrassed (and frustrated) by my failure to grasp the pronunciation of certain words or eat like a proper adult. However, I am inclined to think that their affection for my awkwardness, naivety, and inexperience became a vector through which they could access some *thing* about me. Perhaps my embarrassment and shyness were relatable; maybe they felt similarly during their first days at Tesfa or work abroad? It was, I reasoned, a way in which we could build an understanding about our shared human condition.

Beyond the interpersonal, I also speculate that when someone declined to share their experiences with an outsider, it was associated the aforementioned refusal. They did not want to risk a rekindling of their former conditions through revisited narratives.

Of course, there were also individuals who more freely shared their stories and wanted others to know what happened to them abroad. I am more comfortable conveying their narratives. When I met Kiya, for example, she immediately showed me her hands, craggy with scars. She was forced to use toxic cleaning supplies without protective gloves, worked twenty-hour days, and slept on the floor. She was denied access to clean drinking water and kept hydrated by sipping hot water from the outdoor spigot. All the while she never received compensation.

Another young woman, Taytu, was also very open about her time in the Emirates.

“I experienced something no person ever should,” she said pointedly.

Her female employer constantly berated her and threatened her regularly with a knife, letting her know that the possibility of her death was ever present. One day, while Taytu was washing the skylights, her employer whacked the ladder from beneath her feet. When Taytu hit the floor, her employer kicked her repeatedly, causing a loss of consciousness. Thereafter, she was brought to a Dubai hospital, and later to the *delala’s* house. She recalls that during that time she had a lot of “confusion” and lost her “memories.” A week or so later, she was sent back to Ethiopia and taken to Tesfa.

“I remember the airport nurses carried me here; I didn’t speak for three days.”

## MEANING-CENTERED CARE AND TC

### Meaning-centered care

As I listened to the experiences and witnessed the recoveries of Zeynab, Taytu, and Kiya, I was compelled to consider the efficacies as well as shortcomings of global and local practices of mental health care operating within Tesfa. Beyond the pharmaceutical remedies offered by the nurse, I was intrigued by the ways in which long-term residents responded to newly arrived distressed returnees. Their relaxed attitudes often stood in contrast to my own inner feelings (I was sometimes worried by a newcomer’s behavior). As discussed momentarily, I came to recognize their composures as empathic responses to an issue of concern. Their comportments had meaning; they were rooted within a specific social, cultural, and psychological arrangement attached to domestic work, which yielded a specific knowledge. It was a situated knowledge that drew from personal experience as domestic worker abroad—of feeling and thinking, of performing action and receiving impressions—that generated a distinctive caring response and a valuing of certain behaviors (Held 2006, 23, 27). Their relaxed comportments and remarks of experiential familiarity exemplified an ethics of care that relied upon an empathic understanding of past experience and current presentations of distress.

This orientation led me to consider how such embodied knowledge reflects humanistic and meaning-centered models of mental health care, a practice which anthropologists and some cultural psychiatrists have found common ground in their shared appreciation. Both disciplines, as psychiatrist-anthropologist Ippolytos Kalofonos describes, have endeavored “to understand the meaning and significance of the narratives of people experiencing psychosis,” and, more broadly, distress (2023, 1092). By contextualizing an individual’s distress within distinct social, cultural, political, and historical contexts, anthropologists in particular have argued that causes as well as cures of distress are not only moored to an intersectionality of self but reflect and respond to the presence and accessibility of technological, institutional, and familial interventions (Biehl 2005; Brodwin 2013; Garcia 2010; Giordano 2014; Myers 2024; Nakamura 2017; Sousa 2016).

Kalofonos follows this thread by specifying how clinical practice can build empathy, connection, trust, and therapeutic rapport by advancing a more meaning-centered psychiatric care model that explores the “who” and “what” behind a person’s vulnerability, inequity, marginalization, fear, and pain. This shift in perspective—from asking “what is wrong with you” to “what happened to you”—not only changes the way distress is understood but can also transform the concept of best care by encouraging clinicians to focus on the specific experiences, culture(s), and circumstances that shape an individual’s symptomology (Kalofonos 2023; Luhrmann & Marrow 2016).

Kalofonos’s meaning-centered work also evokes key existential and humanistic psychotherapeutic practices that emphasize meeting a (distressed) person where they are by providing a safe place for the psyche. This practice often involves acknowledging and accepting an individual’s lived realities through empathic, reciprocal, and intercorporeally attentive forms of engagement—approaches recognized as both ethical and effective in mental health care (Bertelsen et al. 2025; Bøe et al. 2024; Eneman et al. 2019; Ghaemi 2007; Havens 1996). Such existential and humanistic orientations resemble the calm, gentle, and everyday interactions I observed within Tesfa, a therapeutic sociality that reflected an intercorporeally attentive, experientially acquainted, and empathic understanding of what had happened, what was happening, and what was needed.

Yet, before returning to my ethnographic data, I want to examine two psychiatric institutions, past and present—Traverse City State Hospital and Bethel House—to show how comparable meaning-centered and humanistic principles shaped effective psychiatric TC in distinct historical and geographic settings. I illustrate that, although each facility is unique, a similar ethics of care—one that embraces the ambiguity of individual distress, centers empathy, and decouples authority from clinicians and pharmaceuticals—operates across both communities. I draw attention to the ways clinicians in both institutions actively cultivate a similar ethics of care through individually responsive practices of respect, reassurance, and even love, rather than relying exclusively on biomedical protocols that tend to stipulate more universalizing approaches to psychiatric care. I then turn to my ethnographic data to show how a comparable process unfolds in Tesfa. There, however, a humanistic ethics of care is not orchestrated by clinicians or mental health professionals. Instead, it is the embodied knowing and experiential familiarity of healed returned domestic workers that precipitates a form of ethical care that is conceivably best fit for newly arrived distressed returnees.

### Therapeutic communities

At a basic level, Traverse City State Hospital and Bethel House exemplify the TC paradigm that first emerged in British military hospitals during World War II as an approach to psychological disorders (Whiteley 2004). Conceptually, TC advanced a distinct social–psychological method of treatment for mental illness and related disorders (and later, addiction) by asserting that individual change must occur through self-help and communal living to render stable modifications of self and the attenuation of socially destructive patterns of behavior (De Leon 1985). Distinct from traditional custodial psychiatric institutions of that period, TC emphasized reciprocal communication, including the discussions of feelings, information sharing, group interaction, problem identification and solving, and learning as a social process. By promoting a more dynamic and relational understanding of distress through open dialogue among members, TC represented a significant departure from the physician-dominated structures characteristic of that era (Jones 1979, 141, 145; Millard 1996).

### Traverse city state hospital

Known at the time for its “moral treatment” and “beauty is therapy” philosophy^3^, Traverse City State Hospital (TCSH) gained state-wide attention following the appointment of Dr. Jack Ferguson in 1954. He was tasked with serving as the primary physician for the hospital’s 1000-bed female ward, which was characterized by “hopeless” and “problem” cases often sent from the more well-known, and well-funded, Ionia State Hospital and University of Michigan Neuropsychiatric Institute. Although initially hired for his expertise in performing the three-minute transorbital lobotomy, upon his arrival to TCSH, he disavowed it, reasoning that it was not worth the trade-offs: The loss of creativity, initiative, and concern for the future (de Kruif 2018 [1957]). His rejection was also based on his own experiences as a psychiatric inpatient—he was grateful that he had not been lobotomized.

Over the following years, Ferguson closely followed the chemical revolution, testing combinations and dosages of groundbreaking drugs (Thorazine, Ritalin, Serpasil, and insulin) and soon earned himself a reputation for successfully engineering pharmaceutical remedies. Yet, he remained skeptical and openly questioned nationally propagated pharmaceutical treatment plans. He cautioned other clinicians that these drugs were limited in their ability to cure, and that “the medicines tend to make us forget that all these patients are human. The way they awake people out of their darkness, they might make you think patients are only chemical” (de Kruif 2018, 174). He maintained that curative psychiatric care required more than drug therapies and needed what he described as, “plain science”: “tender loving care” that recognized each patient as an individual with their own histories, experiences, fears, and sensibilities. To practice this plain science, he encouraged his staff to utilize an empathic approach that emphasized engaged listening and an “egoistic” feeling of what would be best, asserting that he often asked himself, “What would I want if the positions were reversed?” (de Kruif 2018, 179).

Ferguson also insisted on valuing the opacity of each patient’s symptoms rather than supplanting them into predetermined categories and following fixed protocols. He reasoned that patients—much like his previously institutionalized self—were generally misunderstood, and if clinicians wanted to provide the best care, then they must take the time to learn their patients’ modes of expression. He argued that it was only once an understanding was gained about an individual’s situation that their corresponding care—grounded in respect and love—that the clinician could truly succeed in curing mental distress. Despite collegial skepticism, the triumph of his “plain science” was clear. In just two years, the number of women well enough to be discharged to their homes increased by 600 percent, a rate so revolutionary that the University of Michigan and Ionia State Hospital continued to send him their most problematic cases.

### Bethel

Five decades later and six thousand miles away, anthropologist Karen Nakamura (2017) beautifully details the inner workings of Bethel House, a culturally distinct, but philosophically resonant psychiatric community in northern Japan. Initially conceptualized as an “unintentional intentional community,” Bethel mirrors several principles of the TC and meaning-centered models, including its emphasis on moderating the authoritarian position of the psychiatrist, and promoting the collective interpretation of an individual’s illness and everyday struggles. By upholding an individual’s subjective illness experience through affirming, trusting, and sympathetic care—much like Ferguson’s tender, loving, care—Bethel’s “philosophy of nonsupport,” redirects the clinician’s responsibility away from curing a mental illness and toward cultivating a safe and supportive environment that validates the patient’s lived realities.

Nakamura also calls attention to Dr. Kawamura’s (Bethel’s psychiatrist) practice of “therapeutic affirmation” (Kitanaka 2012) through his use of short phrases such as, “you’ve been having a really tough time, huh?” Intentionally employed as a means to acknowledge the patient’s suffering, Kitanaka (2012) and Nakamura (2017) note that such therapeutic affirmations, while simple, tend to serve as a transformative moment for patients, underscoring the remedial qualities of clinical empathy as a sort of, “‘being in touch,’ which allows us [clinicians] to feel the texture of another’s lived world” (Kirmayer 2008, 465). While such statements, and clinical empathy, have their limits and may require ongoing dialogue of accuracy (Hollan 2008), what Dr. Kawamura and Bethel House uphold is an empathically grounded acknowledgement of an individual’s distress and how it articulates in the world, without subsuming it under diagnostic authority.

Though briefly considered, Bethel and TCSH exemplify two culturally and historically distinct TC, which substantiate how healing emerges from a sustained engagement in a communal (social) world structure by validation, reciprocity, respect, and concern for the other—processes that communicate empathic support through meaningful mutual- and self-understanding. While such moments of socially and relationally mediated understandings are evidenced through reciprocal style of communication, the women of Tesfa expand this practice by demonstrating that such understandings can also manifest “beyond words” (Wikan 1992) and through an embodied knowing.

## “BEFORE I WAS THERE, SO I UNDERSTAND HER”

I arrive at Tesfa around ten in the morning. Before I enter the locked compound, I can hear the shouts and rapid speech of a woman. I tap three times on the gate, and my favorite old guard opens the door. We smile at each other, nod, clasp hands, and say our morning pleasantries. There is no mistaking from where the shouts emanate.

A woman stands at the top of the steps leading to the front of the house. She wears an all-black hijab and abaya; it accentuates her face and flows around her figure. Her arms are stretched high above her head while she grasps a leatherbound Quran. I move around the courtyard, greeting other women. Everyone is well and in good spirits.

“Does anyone understand what she is saying?” I inquire.

Everyone shakes their heads; no. One woman, Fanosh, clarifies that she occasionally catches a word in Arabic or Somali (I later learn she is speaking Arabic, and reciting verses from the Quran). Based on her dress and language she speculates that this woman is from the Somali region. I pull a chair alongside Fanosh. Abinet and I make small talk with her against the background noise.

The guard joins our conversation. According to him, this woman—Faduma—arrived last night from Kuwait. The Ethiopian Red Cross ambulance brought her from the airport and dropped her at the front gate around three or four in the morning. The psychiatric nurse is off today, so Faduma has been able to carry on without intervention.

“She has not stopped talking since. No sleep. Five hours nonstop talking,” he tells us.

A Tesfa administrator appears from behind the house. He is touching up some spots of peeling paint along the flowerbeds.

He looks over at our group and calls out, “Fanoooosh! You used to be like this!”

He laughs.

Fanosh smiles shily.

“For one day,” he continues, “and then you started wearing sneakers!”

Everyone erupts into laughter, including Fanosh.

He is right. A short time ago, Fanosh exhibited her own, as she described it, sickness. She repeatedly kicked off her shoes, launching them into the air like little missiles; she pried large rocks from the elevated flowerbeds and attempted to toss them over the compound walls. She tried to snatch a necklace off the cook while she delivered her food and yelled indiscriminately that she killed a person. But like with Faduma, her fellow residents remained composed. They moved out of the way when she stormed through the courtyard. They encouraged her to eat, reassured her that she would be reunited with her family, and invited her to play in their card games. Now, a week later, she is “healed,” free of her earlier symptoms and wearing sneakers.

We continue to chat and watch Faduma stand in her position. Her voice oscillates as she talks ceaselessly. She continues to hold the Quran above her head, but she appears to be tiring. Beads of sweat form across her forehead. She starts to cry. We remark on this as a possible good sign. Maybe she will sleep soon.

She shouts, “Open the heavens door!”

She points in our direction, switching to Amharic, “The devil is in the chair. The devil is writing!”

I am in the chair. I am writing. My whiteness marks me as an outsider, and, in her view, a possible Luciferian interlope.

There are some giggles.

I turn to Fanosh, “What do you think?”

“Before I was there, so I understand her.”

“Are you worried?” I respond.

“No,” she replies.

Registering my own discomfort, I ask, “Are you scared?”

“No.”

Faduma moves down the steps and toward the water spigot. She demands a cup of water from another resident who likewise arrived yesterday. The young woman, draped in a turquoise dress and a patterned hijab, obliges. She brings her a cup of water and squats down beside her. There is some discussion as to whether Faduma asked the other newcomer for a drink because she, too, is Muslim. Faduma sips a bit of water and pours the rest over her head. She tosses the metal cup. She takes a few steps forward. She hits and spits on the guard. He wipes his face as she walks on.

Fanosh’s responses to my questions were not atypical. Her recognition of “before I was there, so I understand her” and relaxed being-with equally reflected the sentiments and composure of Zeynab in my opening vignette. Residents were rarely perplexed or alarmed by such behaviors expressed by distress newcomers. On another occasion, while speaking to the aforementioned Taytu about a trio of new returnees, she drew my attention to one sitting in the corner. She was staring downward, holding her knees tightly against her chest. According to Taytu, this woman would not speak, eat, or move. Raising her eyebrows, she tells me, “I was like that,” offering this information in a can-you-believe-it sort of way. It was hard to believe, as at present Taytu was the most spirited of residents. But that was her point.

Having experienced a similar state, Taytu seemed to understand the newcomer and as such, presented an absence of worry. Recall from her story, she was like *that;* for three days she did not speak. Now, she is like *this*—a precocious teenager who playfully teases me. As I observed Taytu over the next few days, I witnessed this understanding translate into gentle acts of care: bringing the quiet woman a plate of food, encouraging her to eat, but never forcing conversation or movement. Her actions expressed an embodied knowing that granted her an understanding about that which may otherwise be misconstrued, misinterpreted, and responded to in unfitting ways.

## AN ETHICS OF CARE: THERAPEUTIC SOCIALITY

These little remarks from Zeynab, Fanosh, and Taytu were curious to me, as they often occurred in the company of another’s (occasionally disruptive) distress. While I was sometimes startled or made uneasy by someone’s shouting, throwing of objects, or irritated pacing, my interlocutors were—for the most part—unruffled. Their reasons for being so was ostensibly captured by their claims: “Before I was there, so I understand her;” “I was like that;” “I know their feeling.” Each statement expressed a first person-like perspective toward another that involved an emotional, embodied, and experiential knowledge, as well as, I suggest, a future oriented imaginative projection of what was to come (Hollan & Throop 2008, 392; Strauss 2004)—an amalgamation that distinctively marks their empathically oriented ethics of care.

Zeynab, Fanosh, and Taytu were not worried about or scared of the distressed newcomer because they too had once been “sick” and caused no harm; and as such, the newcomer would be the same. They dismissed the witnessed distress as abnormal-and-impenetrable; or as unrelatable and incomprehension. Instead, the newcomer’s distress was positioned as an embodiment of a particular social world, the consequence of migrant domestic work—a familiar sickness.

In positioning the newcomer’s distress as a familiar sickness, residents also captured the assumed temporal dimension: Before I was *there* and *her*—a woman temporarily migrating for a chance at a better life, a domestic worker abroad, a container of mistreatment and subjugation, an eruption of affectivity. Soon she will be *here*, like *me*. “Soon they will calm down,” as Zeynab remarked; she knows this because she did. She, like Taytu and Fanosh, expresses a wisdom of valuing the body and the spaces it has occupied, occupies, and will occupy; it is a knowing that elevates embodiment and the temporal nature of being, wherein one can claim knowledge about an experience and a self—past, present, and future.

As I listened to their words, and watched their interactions, I also came to understand resident’s recognitions of self-sameness, along with their relaxed comportments, as expressions of social, emotional, and cultural knowledge that represented a form of best, good care (Mattingly 2014). Specifically, I considered their calm, gentle, ordinary, and seemingly mundane ways of being-with the distressed newcomer as ethical formations of care, which could be expressed directly or indirectly. Directly, for example, I witnessed residents reassure mute or frightened newcomers through soft, nearly whispered explanations that they were now safe, no harm would come to them here, and soon they would be reunited with their family. The phrase, “*Ayzosh*, *Ayzosh*” (“It is going to be okay”) was a typical response to an upset newcomer. A simple and commonplace Amharic phrase, it is regularly spoken to palliate someone’s feelings of nervousness, stress, sadness, despondency, or fright. Saba explained to me the value of this type of interaction a few days after her (somewhat dramatic) arrival. She recalls her initial frightening encounter with the police and nurses inside the airport.

“I didn’t want to go with them…they were saying ‘You have to go! It is a nice shelter!’ But I was refusing. I was shouting and kicking…And when I arrived at Tesfa that guard got [received] me and he tried to explain to me ‘Your family will come here.’ I was very nervous. I didn’t listen to him because I was very scared. I didn’t sleep. I was crying. All the women were telling me, ‘*Ayzosh*, *Ayzosh*. You need to calm down; you will be reunited with your family soon.’ It was very helpful.”

Like Dr. Kawamura’s “therapeutic affirmations,” women of Tesfa utilize their embodied knowledge to establish a therapeutic sociality through a small, but impactful verbal consolation. Saba’s shouts, thrashing, and crying did not dissuade other residents from approaching and comforting her; they were experientially acquainted with Saba’s condition and knew how to respond best. We saw a similar practice in the opening vignette: A woman attempts to speak with the muted newcomer, she does not reply. Yet, she is not pressured to communicate, nor is she dismissed as alien or unfamiliar. Instead, she is offered a chair so that she may be part of the sociality of Tesfa.

Beyond words, I also witnessed acts of corporeal care that envelop one into the social life of Tesfa. There were moments like Taytu’s gentle guiding of a torpid woman around the courtyard, as well as the wrapping of a damp scarf around the head of a young woman bedridden with a migraine. However, what was most striking to me was the way in which residents simply let a distressed woman be; it was a way of being-with that stood in such contrast to the moments between the distressed newcomer and the psychiatric nurse or providers at the psychiatric hospital—who were quick to inject a loquacious or catatonic woman with an antipsychotic medication or flood an upset returnee with dozens of questions.

In my observation, the women of Tesfa understood how the presence of simply being-with one another with minimal words or amongst the routines of everyday life was itself a meaningful act of care. It was a being-with, a relationality and sociality, that was unbothered by Faduma’s shouts, demands, and hallucinations; a being-with that was non-reactive, reassuring, and did not stigmatize someone’s desire (on inability) to not speak. It was a way in which previously and presently distressed women were simply, but profoundly, “being human together” (Skatvedt 2017). This coexistence was distinctively empathetic, wherein the togetherness between those previously and currently distressed was not dominated by alterity but instead by an embodied knowing of what it was like to have been there. As a mode of being-with, it built and communicated a shared understanding among residents, while simultaneously producing a sense of safety and familiarity, substantiating Hollan’s (2014) claim that embodied forms of attunement are critical to empathy.

The women of Tesfa appeared to know that letting one be, as well as interacting in calm, gentle, and commonplace ways, served as mechanisms for soothing a distressed newcomer. There was an embodied knowing that this type of sociality served as an antidote to their experiences abroad, where their affectivity was kept on edge and their essential humanity perpetually denied (Fanon 2004, 19, 182)—an undertaking Taytu and Kiya’s employers vehemently exemplified through their unrelenting pursuits of emotional and physical torment. Therapeutic sociality strove for the opposite; it worked to reestablish a safe sense of self- and other-awareness by calming one’s affectivity through gentleness and familiarity. The simple, ordinary, and mundane ways in which Tesfa residents responded to the distress newcomer were therefore not trivial, indifferent, or unconcerned, but rather techniques of ethical caring that operationalized the specific needs of a vulnerable other (Held 2006, 23; Tronto 1993).

It is also worth noting that the therapeutic sociality of Tesfa transcended broad cultural differences. I never witnessed dissimilarities amongst women in the shelter (e.g., ethnicity, language, religion)—which are often drawn upon in public and governmental discourses to explicate the contentious relations within Ethiopia—influence how women spoke to and interacted with each other. Someone’s Christian, Muslim, Tigray, Amhara, or Oromo identity was never used as a reason for *not* caring, not understanding, not empathizing. It is such observations that lead me to suggest that women in Tesfa themselves constituted a local culture that oriented the empathic process not along a single shared dimension of culture, religion, language, ethnicity, gender, or even singular experience, but instead along an imagined interiority of the why, how, and what of pursuing domestic work abroad: Why one undertook such a journey, how it felt to encounter the unexpected, and what it meant to get “sick.” Therefore, what helped rehabilitate women in Tesfa was not only being among Ethiopian women (Aziz et al. 2023) but being among their fellow countrywomen who experienced and understood what it meant to go abroad and how it felt to live through the unanticipated violence of domestic work. It is this plural experience and imagined we-ness that constitutes a local variety of empathy and care, further demonstrating that close personal relationships are not necessary for either empathy or ethical care to endure.

## LIMITS OF THERAPEUTIC SOCIALITY

While illustrating how empathy articulates within situations of distress and precipitates a practice of care advantageous to a particular community of women, I also want to recognize that the present evidence draws from moments when women did not feel that their lives were in danger due to the behaviors of a distressed newcomer. Indeed, there were instances during fieldwork when an individual threatened or disrupted the wellbeing of residents and, in such cases, I observed a threshold to empathic understanding.

On one such occasion, an interlocutor became increasingly irritated with a fanatical newcomer. Although she did not say anything directly to this woman, she did huff away from our conversation and proclaimed the newcomer to be, “very annoying!” Another time, during a weekend without the psychiatric nurse, an “aggressive”^4^ newcomer arrived early in the morning. She started a fight with another resident and threatened to kill her. She attempted to go to the kitchen to acquire a knife. Fortunately, the kitchen was locked. The concerned guard made a desperate call to the nurse and director, imploring them to come. After explaining the previous night’s chaos, Kiya said to me, “I was very scared! I said to the guard, ‘why don’t you give me the keys [for the kitchen] and I will sleep inside. When she leaves, I will leave the kitchen.’ The guard was scared too; we were all scared!”

Such situations offer further evidence that personal distress may interfere with one’s capacity for empathy, and that empathic care may require some sense of bidirectional, reciprocal interaction (Hollan 2008; Throop & Zahavi 2020). It is therefore within the same limits that a therapeutic sociality becomes possible, or not—one contingent upon the expression of sickness, the felt assurance that one will not seriously harm another, and a recognition grounded in the sense that “I was like that.” The possibility of a therapeutic sociality thus relies on both a cognitive and experiential reflection that draws on past and future oriented knowledge—of having-been-there and what-will-be.

## CONCLUSION: FROM PAST VULNERABILITY TO A PRACTICAL AND ETHICAL RESOURCE

By highlighting a community of Ethiopian women, who have endured mental and emotional distress in relation to their experiences as migrant domestic workers, I have sought to contribute to our ethnographic explorations concerning the connections amongst empathy, ethics, place, practice, experience, and knowledge (Brown 2010; Hollan & Throop 2008; McKearney 2020; Pedwell 2016). Valuing the more subtle and seemingly mundane ways of being together, I suggest that it is through embodied knowing of having been-there and an imagined what-will-be that distinctively bestows upon formerly distressed women an understanding about ethical and good care.

By applying an emic classification of sickness, my interlocutors also upheld the ambiguity of returned domestic workers’ distress, which allowed healed women to utilize a felt sense—an embodied knowledge—to construct imagined shared experiences necessary for empathic understanding. It is this foregrounding of an imagined being and becoming, specific to undertaking migrant domestic work, which enables women to access knowledge about another’s condition and situation. Accordingly, by positioning sickness as a familiar-ambiguous state of being, empathic understanding of the newcomer was not predicated on having the exact same mental state, feelings, or embodied response (Throop & Zahavi 2020, 289). Rather, an empathic understanding and associated knowledge about how best to interact with and care for a distressed newcomer occurred through a recognition that one’s experiences could map onto another’s such that it precipitates a recognition of shared experiential possibility.

Here again is why a feminist phenomenological perspective is advantageous for understanding the mutable relationship among feelings, practices, and experiences associated with the relational production of empathy (Pedwell 2016; Fielding 2017). It foregoes assumptions about sameness and implications of understanding another—or of being understood—and it allows us to examine the correspondences amongst individuals who “act out of situations,” which helps to locate local varieties of empathy among our more ambiguous and errant ways of being (Beauvoir 2011; Fielding 2017, xvii; Hollan & Throop 2008, 386; Mattingly 2024).

More pragmatically, it is my hope that such experiential knowledge expressed by residents of Tesfa may also work to unsettle the certainty of mental health care paradigms informing Ethiopia’s professional initiatives associated with distressed domestic workers. I am concerned that the rapid assignment of Western DSM/ICD-style psychiatric diagnostic labels and pharmaceutical interventions may contribute to what Timimi describes as the “undermining [of] existing cultural strategies for dealing with distress, more, not less stigma, and the imposition of an individualistic approach that may marginalize family and community resources, and [divert] attention from social injustice” (2014, 212). What the women of Tesfa prove is that embodied knowledge about what constitutes good care was not only effective in ameliorating numerous symptoms among distressed returnees but also helped to avoid further distress. Women of Tesfa illuminate important cultural configurations of empathic care that warrant further consideration as an advantageous practice of care for places of first arrival—airports, rehabilitation homes, and the psychiatric hospital. Indeed, it is imaginable that, upon return to Ethiopia following abusive and traumatic experiences abroad, efforts to calm one’s affectivity might benefit reintegration and rehabilitation efforts.

Although I am not suggesting that therapeutic sociality replace the formal and institutionalized treatments offered by clinicians or the psychiatric hospitals in Ethiopia, I am proposing it as a possible first-line therapy. My proposition is not only based upon ethnographic and observational evidence, but also considers recent calls from Ethiopian researchers for culturally appropriate psychosocial intervention to address distress among migrant returnees in Ethiopia (Desie et al. 2024), as well as the documented success of community-run configurations of empathic psychosocial care, such as Chibanda’s “friendship bench” in Zimbabwe and “talking is mental” in Scotland (Carmichael 2025; Chibanda et al. 2011; McFadden 2025). These interventions offer substantial benefits through the establishment of familiar, stigma-free environments—places where one’s psyche can safely (re)engage with the external world.

The therapeutic sociality of Tesfa provides a concrete model for how such first-line care might operate. As evidenced by Zayneb, Fanosh, and Taytu, such seemingly mundane remarks, ordinary routines, and gentle gestures are more than mere pleasantries. They constitute an ethics of care through which previously distressed women transform their own experiences of suffering and past vulnerability into both a practical and suitable resource for recognizing, interpreting, and responding to the distress of others. In doing so, they exemplify what it means to be empathic and offer care in the midst of difficult and messy situations.

## Footnotes

1 *Delala* can mean “agent” or “broker”; it is the person who facilitates the movement and employment of Ethiopian domestic workers.

2 Pseudonym.

3 TCSH forbade the use of restraints, and many residents worked on campus creating a self-sustaining community including farming and livestock production.

4 This is the term Tesfa’s nurse later used to describe her.
